# Older women’s perceptions of HPV self-sampling and HPV-sampling performed by a midwife – a phenomenographic study

**DOI:** 10.1186/s12889-024-17723-7

**Published:** 2024-01-17

**Authors:** Maria Hälleberg Nyman, Gabriella Lillsunde Larsson, Karin Blomberg, Agneta Schröder

**Affiliations:** 1 Faculty of Medicine and Health, School of Health Sciences, Örebro University, Örebro, Sweden; 2 University Health Care Research Center, Faculty of Medicine and Health, Örebro University, Örebro, Sweden; 3 Department of Laboratory Medicine, Faculty of Medicine and Health, Örebro University, Örebro, Sweden; 4 Department of Health Science, Faculty of Health, Care and Nursing, Norwegian University of Science and Technology (NTNU), Gjövik, Norway

**Keywords:** Cervical screening, HPV, Human papillomavirus, Qualitative, Self-sampling

## Abstract

**Background:**

Cervical cancer is a global disease and it is well established that cervical cancer is caused by human papillomavirus (HPV). In Sweden self-sampling for HPV is now used as a complement to sampling performed by a midwife. However, there is a lack of knowledge on how older women perceive the self-sampling compared to the sampling performed by a midwife. Therefore, the aim of the study was to describe how women, aged 64 years and older, perceived the process of self-sampling and sampling performed by a midwife for HPV-testing.

**Methods:**

Eighteen women were included in a qualitative interview study, and a phenomenographic approach was used for the analysis of the interviews.

**Results:**

Three descriptive categories emerged: Confidence in sampling, Facilitating participation and Being informed. Within the categories, eight conceptions emerged describing the variation relating to how the women perceived the process of self-sampling and sampling performed by a midwife.

**Conclusions:**

Women in this study describe confidence in self-sampling for HPV-testing and that the self-sampling was saving time and money, both for themselves and for society. Information in relation to an HPV-positive test result is of importance and it must be kept in mind that women affected by HPV may feel guilt and shame, which health care professionals should pay attention to. This knowledge can be used in education of health care staff.

**Trial registration:**

https://researchweb.org/is/fourol/project/228071. Reg. no 228,071.

## Background

Cervical cancer is a global disease and the third most common form of cancer among women. In 2018, 569,000 women were diagnosed, and 311,000 women died from the disease [1]. This is an undisputable fact, despite available primary prevention such as HPV-vaccination and screening for precursor lesions as secondary prevention.

It is well established that cervical cancer is caused by human papillomavirus (HPV) [2, 3]. An HPV infection is a very common gynaecological infection that most often clears without intervention. If the infection becomes persistent, there is an increased risk for lesion development and progression to cancer [4]. Many different HPV genotypes have been described, but it is high risk (hr) HPV that has the potential to introduce lesions that can develop into cancer. Thirteen HPV genotypes are in the hrHPV group with the highest risk score for cervical cancer development [5, 6].

In Sweden, there has been a national screening programme for cervical cancer since the 1960s. However, despite the success in reducing the disease, approximately 550 women still get it each year [7]. The method of choice in screening for cervical cancer has recently changed, and primary hrHPV-testing is now replacing cytology in screening algorithms in many countries, including Sweden. Sampling for HPV-testing in Sweden today is mostly performed by midwives in the health care setting, with some exceptions, such as reaching non-attenders. The current screening algorithm in Sweden today includes primary HPV-testing för women 23–70 years where women 23–49 should be tested every 5th year and women between 50 and 70 every 7th year. In the new guidelines for screening in Sweden, self-sampling is also suggested as an alternative for all women if requested. Recommended triage test after a positive HPV result is cytology [8].

Screening with HPV-testing can lead to improved follow-up for both younger and older women. Cytology testing in the postmenopausal age group has shown low sensitivity and specificity for detection of cervical dysplasia [9, 10], and this can thus be improved with HPV-testing. This is important since the Swedish screening programme now includes women up to 70 years of age [11]. Also, when using HPV-testing, professional sampling by a midwife can be replaced by vaginal self-sampling. Self-sampling for HPV-testing has been proven to be as good as or better than professional sampling in detecting high-grade squamous intraepithelial lesion (HSIL) and cervical cancer [12, 13] and can also increase participation among non-attenders [12, 14–16].

Attendance of screenings, regardless of screening method, depends on the adherence to invitations to participate. Earlier studies describe flexibility and own choice of time and place for testing to be important factors that could facilitate attendance. Other factors described are the importance of sufficient information on the relevance of testing and on HPV as a cause for cancer [17, 18]. Qualitative studies that focused on self-sampling as a method of choice reported self-testing to be less embarrassing and less uncomfortable compared to professional sampling, as well as less physically and emotionally discomforting [19, 20] and less time-consuming [21].

As mentioned before, self-sampling is already used in the Swedish screening setting for long-time non-attenders [11], but may potentially also be a first-choice alternative for all women in the future, depending on new triage testing methods for HPV-positive women. However, with an increased screening age, studies are needed to further investigate the acceptance and confidence of self-sampling versus professional sampling in women ≥ 60 years of age. Understanding the acceptance also in the older age group is important because about 30% of cervical cancer cases occurs in women older than 60 years of age and mortality is also high in this group [7, 22].

## Aim

The aim of the study was to describe how women, aged 64 years and older, perceived the process of self-sampling and sampling performed by a midwife for HPV-testing.

## Method

### Design

This study has a descriptive qualitative design with a phenomenographic approach, attempting to identify the different ways people perceive and understand phenomena in the world around them [23, 24]. Phenomenography makes a distinction between the first-order perspective of what something is and the second-order perspective of how something is perceived [25]. The essential factor in phenomenography is the second-order perspective [25]. In phenomenography conceptions are central to understanding a phenomenon and have their origins in individual interviews but the results are a description on a collective level [25].

This study was a part of a lager study with the aim to evaluate and compare vaginal self-sampling to professional sampling for HPV-testing among women aged 64–69 years in one county in the middle of Sweden. A total of 7,835 women were invited to participate in a catch-up screening between 2018 and 2020. They were in parallel invited to participate in the overall study and among those, 2,258 accepted. A sub-section of the total study population was selected to participate in the current study.

### Sample and settings

The selection criteria were that the participants had to have participated in the overall study on sampling methods and performed one or both testing methods (vaginal self-sampling and/or professional sampling by a midwife) during 2018–2020 in a county in the middle of Sweden. The selection criteria were purposeful in that they were guided by the desire to find participants who varied in their perceptions of the different sampling methods. Women’s vaginal self-sample and professional sample were both tested for HPV using the same screening method for HPV detection (Aptima, Hologic) that detects mRNA from 14 different hrHPV genotypes.

A subset of 20 women who had agreed to be part of the overall study were contacted first by mail and later by telephone for initial information of the study and were asked if they were willing to participate. If they agreed to participate in further interviews, a time for the interview was booked.

The final sample consists of 18 women from both urban and rural areas, differing in age, educational level, occupation and working life status (Table 1).

**Table 1:** Characteristics of participants

Total		(n)18
Age, range (years)		64–69
Education level (highest)		
	Primary school (7 years, ‘*folkskola*’)	1
	Compulsory school (9 years, ‘*grundskola*’)	1
	Upper secondary school (12 years)	9
	Undergraduate school (15 years)	6
	Missing	1
Previous occupation	Health care related*	10
	Non-health care related	8
Working life status	In employment	1
	Retired	16
	Missing	1

*Registered nurse, licensed practice nurse, occupational therapist, rehabilitation staff, social worker, laboratory assistant

Table 1 is showing the education level and working life characteristics of the participating women.

The study was approved by the Regional Ethical Authority in Uppsala, Sweden (reg no; blinded for review).

All participants were legally competent to give their consent. Participation was voluntary and could be terminated at any time, and confidentiality was assured.

### Data collection

The interviews were performed by a psychiatric nurse and researcher with experience in qualitative methods, between June 2019 and June 2020. The interview guide used in all interviews was developed by the last author and the researcher performing the interviews in collaboration with the researchers responsible for the main study, who are experts within the HPV field.

The main question was: (1) Can you describe how you perceive your acceptance of self-sampling versus professional sampling during cervical cancer screening? (2) Can you describe how you perceive your confidence in self-sampling versus professional sampling in cervical cancer screening? Follow-up questions were asked to initiate further accounts. The number of follow-up questions depended on how fully and precisely the participants answered the main question. The interviews were conducted in the form of conversations in the participants’ homes or in a room at the research centre, except for 7 interviews which were conducted via telephone because of the COVID-19 pandemic. During the interview, the participants filled in a consent form. The interviews took between 7 and 20 min (in total 4 h), were audio-taped and later transcribed verbatim by a professional transcriber.

### Data analysis

Phenomenographic analysis focuses on similarities and differences between individual statements, moving from an individual to a collective consciousness as a “pool of meaning” (25). To enable greater familiarity with the data, the analysis was done manually by MHN, GLL and AS. Two of them were knowledgeable in qualitative methods. The analysis was carried out in four phases [24, 26, 27].

In the first phase, the recording was listened to alongside the transcribed text in order to make sure that the interviews were correctly transcribed. Thereafter, the whole text was read a number of times with an open mind to get to know the data. Notes were taken and statements relevant to the aim of the study were identified to find an answer to the following question: What are the different ways of perceiving the phenomenon (vaginal self-sampling versus professional-sampling)?

The second phase consisted of identifying variation, that is, similarities and differences between the ways the informants described the phenomenon. The essential part of the analysis was the comparison of significant statements. Distinct statements were labelled (coded), and from these labels preliminary conceptions were designed to catch what the participants perceived about vaginal self-sampling versus professional sampling.

In the third phase, to obtain an overall map of how these similarities and differences could be linked, the significant conceptions were compared with one another. Preliminary descriptive categories emerged and were labelled based on findings of appropriate linguistic expressions.

In the fourth phase, the focus was on the relations between the preliminary descriptive categories [24] which were compared to ensure that each descriptive category was mutually exclusive and at the same level.

These descriptive categories were critically scrutinized in order to check that they were in agreement with and represented the conception. Finally, three descriptive categories emerged as the main findings from the interviews. The researcher (KB) who wasn´t involved in the primary analysis reviewed the analysis in order to increase the trustworthiness. There was a continuous interplay between the phases in the analysis, and the four researchers carefully discussed the descriptive categories and conceptions to find consensus, in line with Bruce et al. [28]. The results are presented in an outcome space with a horizontal structure [29] (Table 2). Quotes are represented with codes, indicating the consecutive number for each interview.

**Table 2:** The results presented in the outcome

Descriptive categories	Confidence in sampling			Facilitating participation			Being informed	
Conceptions	I can do it myself	I can do it myself, but	The midwife can do it	Comfort	Saving time and money	Logistics	Information on how to perform the self-sample	Lacking knowledge regarding HPV

Table 2 summarizes the results found in the study where three descriptive categories were found.

## Results

Three descriptive categories and eight conceptions describing the variation emerged relating to how the women perceived the process of self-sampling and sampling performed by a midwife for HPV-testing. The conceptions are illustrated with quotations from interviews to increase trustworthiness.

### Confidence in sampling

This descriptive category brings together three conceptions: *I can do it myself*, *I can do it myself, but* and *The midwife can do it* describing the variation in the how the women perceived confidence in sampling for HPV-testing.

#### I can do it myself

The women perceived they had confidence in themselves in performing the self-sampling. Even though they had never performed HPV self-sampling before, they wanted to learn and were sure they would be able to do it correctly. When they got a test result that was the same as the sample taken by the midwife, it confirmed their perception of self-efficacy. The women described knowing their body and that it felt good to be allowed to do the sampling themselves.

*As women, we are used to taking good care of that part of our body and so there is nothing odd about it. (Woman 4)*

#### I can do it myself, but

The women described how they were able to perform the self-sampling, but they did not feel sure that they had done it right. As they were not able to look into the vagina when taking the sample, they could not see where they swabbed. They expressed that they did not know if the test was ‘fool-proof’ or whether it was possible to do it in a wrong way. If they got a different result on the self-test compared to the test taken by the midwife, they doubted their ability even more. To overcome this insecurity, it was suggested that self-sampling could be performed every other time, having the midwife perform the test the other times.

*Maybe you shouldn’t take it [the HPV-test] all by yourself every time, but maybe you do it every other time or something like that, I do think that a midwife is better at taking the test than I am. (Woman 1)*

#### The midwife can do it

Overall, the women expressed great trust in the midwives, both in performing the sampling and in general. They appreciated getting to meet the midwife, having a personal encounter, and being able to ask other questions regarding vaginal health. The women perceived the midwives as professional and highly skilled, and with great experience of taking screening tests and being able to actually see if there was anything else that is not right in the vagina at the same time as they were taking the sample for HPV analysis.

*Yes, the benefits are, when you think of it, that they (the midwife or gynaecologist) will notice if there are any deviations, I think, I don’t know. And that there is a human being you can talk to if you have any queries, that’s it. (Woman 16)*

The women emphasized that not all women are able to perform a self-sample and for them it is of utmost importance that the opportunity to go to the midwife for HPV-screening must continue to be available.

### Facilitating participation

This descriptive category brings together three conceptions: *Comfort*, *Saving time and money* and *Logistics*, illustrating how self-sampling can facilitate participation.

#### Comfort

Self-sampling was described as comfortable; you could perform it lying in your own bed or in the bathroom. Many women had experienced the feeling of being exposed and vulnerable in the gynaecological examination chair, lying with their legs widely spread. They expressed how they had to ignore these feelings and still go to their appointment at the clinic to take the test. There were also experiences of having a male midwife taking the test, which added to the feeling of being exposed and uncomfortable. By performing a self-sample you could take part in the screening programme but still feel safe and comfortable. The women described that the sample taken by a midwife often could be painful but that the self-sample did not hurt at all, which was an advantage.

*For me it [self-sampling] is a great advantage as you don’t have to, that it is much more uncomfortable to lie in that chair and you tense up, you know, so no, I think self-sampling is an advantage. (Woman 11)*

#### Saving time and money

The women expressed two different perspectives of saving time and money– their own and that of society.

Not having to travel to the midwife reception was timesaving and travel could also be associated with costs, especially if the midwife reception was located far from the woman’s home.

I must say that it’s a bit better to take the test it at home. It saves time, time and money for me who has to travel to the midwife. And before when I was working I had to take time off to go to the midwife. (Woman 15)

They described it as valuable to not have to catch an appointment and be able to take the test when and where it suited them best.

From a societal perspective, they could see that self-sampling could be a way to economize society’s resources, as the midwives could focus on other things than performing all these screening samples. Self-sampling was also seen as an opportunity to remain in the screening programme also at older ages.

#### Logistics

The HPV self-sampling kits were sent home to the women and they perceived it as convenient and a good reminder. When they received the envelope they took the test, put it in the reply envelope and posted it. They could see logistic problems such as the risk of not posting the sample within a reasonable time, or where to find a mailbox. They also identified a risk that the test kit could get lost in the mail, either when sent out to the women or when it was returned.

*No, I think it worked well, it was easy, it arrived by mail, and you sent it off. There were no worries. (Woman 8)*

### Being informed

This descriptive category brings together two conceptions: *Information on how to perform the self-sample* and *Lacking knowledge regarding HPV*, illustrating the women’s perceptions on information and knowledge.

#### Information on how to perform the self-sample

All women received a leaflet with instructions on how to perform the self-sampling, and they perceived it as clear and easy to understand. They described how they read the instructions several times and looked at the pictures in the leaflet to be sure of how to perform the self-sample. Some women also got verbal instructions from a midwife, which was experienced as pedagogical and informative. However, there were some thoughts on how deep in the vagina they should take the self-sample and whether it would hurt.

*There were thorough instructions on how to perform the sampling, otherwise I would have felt insecure. (Woman 2)*

#### Lacking knowledge regarding HPV

The women perceived that they were lacking knowledge in an array of dimensions regarding HPV and HPV-testing. They lacked knowledge on the change in screening method in the Swedish screening programme, leading to questions on how they could have a positive HPV-test now when they always had had negative results on the cytology tests before. When getting a positive HPV-test, the women described an insecurity regarding whether a positive HPV-test indicated having cancer or having pre-cancer. Having different test results on the self-sample (from the vagina) and the test taken by the midwife (from the cervix) made the women feel even more confused. Some women perceived that they did not know who to contact to get an answer to their questions, as there were no phone numbers to call on the letter with the test result. They lacked knowledge on how they had contracted HPV and they were told that they could have had it for a very long time. They also reasoned on whether an HPV infection had any relevance for a woman of their age.

*Because I had two different answers within a period of 2 weeks… and I want to have an explanation if it could have cleared during this time or if it is something you still have, but that it got lost in the sampling. (Woman 3)*

## Discussion

An HPV-based cervical screening programme using self-samples would benefit both younger and older women. To maximize attendance, compliance needs to be investigated in relation to women’s perceptions and experiences of sampling and sampling results. Focusing on older women’s perceptions of the process of self-sampling and sampling performed by a midwife, this study revealed both positive and negative conceptions.

One important finding from this study is the older women’s confidence in self-sampling, with the help of good instructions. It was evident that they knew their own body and that self-sampling could be done easily without the need to visit a health care centre. The majority of the interviewed women had worked in health care-related professions and this might explain their confidence in sampling. Interestingly, this confidence was less prominent when test results from self-sampling and sampling by the midwife were not the same. They then doubted their own ability and not the midwife’s. Within the overall study, wherein the interviews were a sub-study, clinical follow-up was done on any HPV-result from either of the two sampling methods in accordance with the national guidelines for screening. HPV incidence in the overall study differed between the two sampling methods (data not published), with the highest HPV occurrence in self-sample tests compared to the professionally collected tests, a finding that also has been described in other Swedish settings using the same HPV screening analysis [30]. Hence, a difference in results may not necessarily be related to the women’s sampling but to other methodological issues.

The women also described the value of having the professional midwife present to ask questions and potentially also detect other deviations, not visible to the women themselves. This trust in health care professionals has previously been described [21] and is important. Also, it was seen in the interviews that the women discussed having every other test as a self-test and every other one taken by the midwife. This might be an approach to integrate self-care and intrapersonal trust, but also the trust in the health care professionals as experts.

Flexibility in regard to when and where to be tested has been important in previous studies to facilitate attendance [17, 18]. This was also evident in the current study, thus supporting self-sampling as a smart and comfortable choice. Interestingly, the interviewed women reflected on this from both their own and a societal perspective. They described self-sampling as cost-effective and enabling midwives to instead do other things besides sampling for screening. This reasoning can again relate to the fact that most participating women were previous health care-related professionals. Studies on health economics report self-sampling for HPV-testing to be more cost-effective than clinician-collected samples [31, 32], but findings may also depend on triage testing and screening intervals. Future studies on cost-effectiveness in self-sampling need to consider this age group of women as well, now included in the Swedish screening programme, using self-sampling as an exit test for leaving the screening programme.

One other important aspect from the interviews was vulnerability. In self-sampling, the woman does the examination herself and there is no stress in relation to getting into a gynaecological chair or being examined be a man. This data is in line with previous research [19, 20] and might be even more relevant in this age group. For older women there could also be physical challenges getting into the gynaecological chair. In the interviews, the importance of ease and comfort in sampling was also clearly described by the women.

It emerged that the women lacked knowledge about HPV, not knowing it is a sexually transmitted infection and being told that they could have had the infection for a long time. Learning that you have a sexually transmitted infection when you are in your seventies could come as a surprise. This could evoke feelings of guilt and shame described in earlier research on HPV [33]; in particular, concerns about disclosing results to their partner and worries about being judged as being promiscuous were highlighted. Further, there are studies describing women with HPV who, having had the same sexual partner for a long time, could accuse their partner of having cheated on them [18, 34].

Even though the women had been taking part in the screening programme for a long time, it was not clear to them that HPV is the cause of cervical cancer and that previous tests were based on looking for dysplasia in cervical cells instead of the virus itself. It is essential to explore women’s understanding and knowledge of treatment options and their potential thoughts on benefits and risks as well as their values/preferences, as these are significant factors in the decision-making process [35]. A change of screening tests can be difficult to communicate to the public and may raise many questions. Clear informative text in relation to test results are of utmost importance, together with contact information to health care. Although much information is easily accessible nowadays, this was clearly desired by the interviewed women and may reflect this specific age group.

### Limitations

Some limitations of this study should be noted. First, for some women the time span from performing the self-sampling test to the time of the interview was up to a year. There were individual participants who had trouble remembering the details of the self-sampling performance due to the time span, while others could describe the process in detail. However, the variation in the time span between the self-sampling and the interview could be seen as a strategy to achieve maximum sampling variation [36]. We had the intention to include women with different cultural backgrounds and from different countries, but only women fluent in Swedish participated in the main HPV self-sampling study from which we recruited the participants for this interview study. This could affect the transferability to groups with migratory and other cultural backgrounds. Other strategies for recruiting participants to HPV self-sampling studies, such as using community champions, have proved to be effective to reach women who normally do not attend the screening programme [37].

Another potential limitation is that due to restrictions caused by the COVID-19 pandemic, this study used both face-to-face and telephone interviews. In qualitative research, the face-to-face interview has been seen as the golden standard, but a study where Saarijärvi and Bratt [38] compared findings from video, face-to-face and telephone interviews showed that the content did not differ between the different interview methods.

## Conclusions

We conclude from this study that the interviewed women describe confidence in self-sampling for HPV-testing from their own perspective and from a societal perspective. Professional sampling by midwives can complement self-sampling when needed. Information in relation to an HPV-positive test result is of importance and it must be kept in mind that women affected by HPV may feel guilt and shame, which is something that the health care professionals should pay attention to. This knowledge can be used in education of health care staff and provide an educational challenge for society when changing screening tests.

## Data Availability

The data are not publicly available due to privacy or ethical restrictions. Please contact the corresponding author; gabriella.lillsunde-larsson@oru.se regarding requests of original data.

## Abbreviations

COVID: 19–Coronavirus Disease 2019
HPV: human papillomavirus
hr HPV: high risk HPV
HSIL: high–grade squamous intraepithelial lesion
